# Genomic characterization and molecular predictive biomarkers for chemotherapy in patients with metastatic triple-negative breast cancer treated in a real-world setting

**DOI:** 10.1016/j.breast.2025.103874

**Published:** 2025-01-02

**Authors:** Erik Olsson, Henrik Lindman, Evangelos Digkas, Viktoria Thurfjell, Haidar Mir Ali, Ute Krüger, Anna-Karin Wennstig, Marie Sundqvist, Antonios Valachis

**Affiliations:** a Department of Oncology, Uppsala University Hospital, Sweden; b Department of Clinical Pathology, Uppsala University Hospital, Sweden; c Department of Surgery, Kalmar Hospital, Sweden; d Department of Clinical Pathology, Kalmar Hospital, Sweden; e Department of Oncology, Sundsvall Hospital, Sweden; f Department of Oncology, Örebro University Hospital, Sweden

## Abstract

**Purpose:**

We aimed to characterize genomic alterations with potential prognostic or predictive significance in patients with metastatic triple-negative breast cancer (mTNBC) treated with chemotherapy in a real-world setting.

**Patients and methods:**

Next-generation sequencing with FoundationOne® CDx was conducted primarily on primary tumor tissue from 112 consecutive patients with mTNBC. Genomic alterations were subdivided into canonical oncogenic pathways and noted for their involvement in homologous recombination deficiency (HRD). Altered genes and pathways were correlated with overall survival (OS) and evaluated regarding their association with real-world progression-free survival (rwPFS) in patients treated with different chemotherapy agents. Occurrence of alterations were compared between patients with exceptional response and rapid progression to chemotherapy.

**Results:**

After exclusion due to insufficient tumor tissue or clinical data, material from 97 patients was analyzed. The most frequently altered genes were TP53 (82 %), RAD21 (25 %) and PIK3CA (23 %). Altogether, 26 % of patients had an alteration leading to HRD. None of the analyzed alterations were associated with OS. Variants leading to HRD were associated with a prolonged rwPFS in patients treated with platinum-based chemotherapy in the first line setting (hazard ratio [HR], 0.31 [95 % CI: 0.12–0.84]). Exceptional responders more often exhibited alterations in the MYC and RAS/RTK pathways compared to rapid progressors.

**Conclusions:**

Patients with tumor alterations in HRD-related genes seem to define subgroups that respond favorably to platinum-based chemotherapy. Further research into the genomic landscape of tumors from patients with rapid progression or exceptional response to different treatment strategies can provide insights into mechanisms of resistance and identify predictive biomarkers.

## Highlights

• Tumor BRCA mutations seem to be predictive for response to platinum-based chemotherapy in metastatic triple-negative breast cancer.
• None of the most frequently altered genes in metastatic triple-negative breast cancer, including TP53 are associated with overall survival.

## 1 Introduction

Triple-negative breast cancer (TNBC) accounts for 10–15 % of all breast cancer cases [1] and carries a higher risk of relapse and metastasis than breast cancer subtypes with other immunohistochemistry features.

Given the lack of predictive biomarkers, the only available systemic treatment for metastatic TNBC (mTNBC) up until recently has been chemotherapy. However, recent advances have resulted in new biomarker-guided systemic therapies such as checkpoint inhibitors for patients with PD-L1-positive tumors [2,3] and poly ADP ribose polymerase (PARP) inhibitors for patients harboring a germline BRCA mutation (gBRCA) [4,5]. However, chemotherapy remains the backbone for treatment of mTNBC despite the lack of biomarkers guiding the choice of chemotherapy agent and predicting prognosis.

Although mTNBC is proven to be a heterogenous disease in terms of its clinical course and genomic background [6], data on molecular prognostic biomarkers are scarce and conflicting. TP53 mutations have been associated with worse outcomes in some studies including mTNBC but not in others [[7], [8], [9], [10], [11], [12]]. The potential impact of other frequent genomic alterations such as PIK3CA and tumor BRCA mutations (tBRCA) are scarce [11,[13], [14], [15], [16], [17]]. A potential prognostic role of alterations in canonical signaling pathways such as MYC and cell cycle has also been suggested, but with limited evidence [7,18].

Regarding predictive biomarkers for patients with mTNBC treated with chemotherapy, gBRCA has been shown to have a predictive value for platinum-based chemotherapy [19]. However, since a significant proportion of all tBRCA are somatically acquired [20,21], the role of tBRCA as a predictor for platinum-based chemotherapy is not well established although some studies support this potential association [19,22]. Apart from BRCA-mutations, it is not established how mutations in other genes leading to homologous recombination deficiency (HRD) affect treatment outcomes with platinum-based chemotherapy since available data are conflicting and definitions of HRD vary [19,[22], [23], [24]].

An approach to identify potential predictive treatment biomarkers is to focus on exceptional responders and rapid progressors to specific treatment strategies. Based on results from cancer types other than mTNBC, molecular characteristics of tumors from exceptional responders to chemotherapy include aberrant DNA damage response and improvement in anti-tumor immune response [25,26]. However, studies investigating molecular characteristics of exceptional responders to chemotherapy in mTNBC patients are lacking.

As a result, considering the limited and conflicting evidence on prognostic and predictive tumor molecular biomarkers in breast cancer patients with mTNBC treated with chemotherapy, we aimed to characterize the genomic landscape in a real-world cohort of patients with mTNBC focusing on identifying genomic alterations of potential prognostic significance or with predictive value regarding chemotherapy. In addition, we aimed to characterize the genomic background of tumors from patients with exceptional response or rapid progression to chemotherapy.

## 2 Methods

This study is reported in accordance with the ESMO-GROW reporting guidance for oncology real-world studies [27].

### 2.1 Study design and research question

We conducted an explorative, retrospective cohort study of consecutive patients with advanced TNBC (defined as either mTNBC or locally advanced disease not amenable to curative treatment; both referred to as mTNBC). Our research aims were to elucidate:

• Prevalence and type of genomic alterations.
• Any association between common genomic alterations and overall survival (OS).
• Any association between common genomic alterations and real-world progression-free survival (rwPFS) during treatment with taxane-, anthracycline- and platinum-based chemotherapy in the 1st line setting.
• Any association between common genomic alterations and real-world response rate (RR) during treatment with taxane-, anthracycline-, platinum- and Capecitabine-based chemotherapy in any treatment line.
• Any clinical and genomic differences between patients who exhibit an exceptional response or rapid progression on chemotherapy.

### 2.2 Patient cohort and data sources

Patients in the study cohort were derived from four Swedish hospitals that were treated for mTNBC between January 2009 and February 2019.

TNBC was defined as ER expression <10 %, PR expression <10 % and HER2 = 0, 1+ or 2+ on immunohistochemistry staining and without amplification on in situ hybridization in the case of 2+. Patients were treated according to standard of care and response evaluation carried out according to standard clinical practice which reflects real-world assessment of treatment effectiveness.

Biopsies to verify metastatic relapse were performed at the clinician's discretion.

Clinical data regarding patient age at diagnosis of early and mTNBC, tumor characteristics, Charlson comorbidity index [28], treatments, response evaluation, date of death or last follow-up and cause of death were extracted by the investigators manually from electronic medical records or local databases between January 2020 and December 2020.

### 2.3 Genomic analysis

Genomic profiling with Next-Generation Sequencing (NGS) was conducted between September 2021 and January 2023 on DNA isolated from archived formalin-fixed paraffin embedded (FFPE) tumor tissue using the FoundationOne CDx (F1CDx) panel, which is designed to detect substitutions, indels, and copy number alterations in 324 genes, as well as select gene rearrangements and genomic signatures such as microsatellite instability and tumor mutational burden. Genomic profiling of the patients’ germline DNA to exclude potential germline variants was not carried out within the framework of the study, as the majority of patients were deceased at the time of data collection and the fact that F1CDx uses computational approaches to distinguish somatic from germline alterations [29]. Data from germline testing done as part of routine clinical practice were not cellected.

All available tumor tissue samples for each patient (including primary tumors, lymph node metastases, local relapses, and metastases) were identified and evaluated for their suitability for genomic profiling by a clinical pathologist. Tumor samples where tissue quantity was insufficient for DNA extraction or where tumor cell density did not exceed 20 % were excluded from NGS analysis.

Genomic alterations were subdivided into short variants, amplifications, homozygous deletions, and functional rearrangements. Variants of unknown significance were excluded. Altered genes were subdivided into oncogenic signaling pathways according to Sanchez-Vega et al. [18]. HRD was defined as an alteration in any of the following genes specified by Heeke et al. [30]: *ARID1A*, *ATM*, *ATRX*, *BAP1*, *BARD1*, *BLM*, *BRCA1/2*, *BRIP1*, *CHEK1/2*, *FANCA/C/D2/E/F/G/L*, *MRE11A*, *NBN*, *PALB2*, *RAD50*, *RAD51*, *RAD51B*, or *WRN*.

Since metastatic tissue samples were lacking for a significant amount of patients and available metastatic tissue samples frequently were of insufficient quality or quantity for NGS analysis, we decided to use the molecular data generated from the NGS analyses of primary tumor tissue samples for our descriptive, prognostic, and predictive analyses, even when genomic data from metastatic tissue samples were available.

### 2.4 Definitions and outcomes

Real-world RR was defined as the ratio of patients exhibiting either a partial or complete response based on an assessment of clinical and/or laboratory and/or radiological data by the treating physician, to the total number of patients receiving the given treatment.

rwPFS was defined as the length of time from initiation of a treatment to a real-world progression event (based on physician assessment in real-world setting) or death, whichever occurred first. Patients lost to follow-up or who did not experience an event at the data cutoff were censored.

OS was defined as the time from diagnosis of mTNBC to death from any cause. Patients who were alive at the data cutoff or lost to follow-up where censored at the last follow-up.

Exceptional responders were defined as patients with a rwPFS of at least three times the median rwPFS or OS of 5 years or more for mTNBC. Rapid progressors were defined as patients with progressive disease at first evaluation to two consecutive treatment lines (or to first line if no subsequent treatment line was given).

tBRCA was defined as a likely pathogenic or pathogenic BRCA1/2 mutation in the tumor tissue regardless of whether it was somatically acquired or germline derived.

### 2.5 Statistical analyses

Due to the exploratory, hypothesis-generating nature of the study no formal sample size calculation was performed.

Since all patients’ tumor samples were not suitable for NGS analysis, only patients where NGS data and clinical data on treatments and responses were present were included in the descriptive, prognostic, and predictive analyses.

All clinical characteristics and treatment patterns were summarized using descriptive statistics.

Frequencies and patterns of genomic alterations were visualized through the Oviz-Bio Landscape application [31].

The association between genomic alterations and OS was explored through univariate Cox proportional hazards regression models for calculating hazard ratios (HR) and corresponding 95 % confidence intervals (CI). For genomic alterations with potential prognostic value (p-value <0.05) in univariate analysis, a multivariate Cox regression model adjusted for well-documented prognostic factors was planned.

The predictive role of genomic alterations based on different chemotherapeutic strategies (taxane-based, anthracycline-based, platinum-based) was analyzed using real-world RR and rwPFS. For real-world RR, univariate logistic regression models were utilized to calculate Odds Ratios (OR) and corresponding 95 % CIs for specific genomic alterations within each treatment strategy. For rwPFS, univariate Cox regression models were employed to calculate HR and corresponding 95 % CIs within each treatment strategy. Due to the low number of patients in each treatment group, no adjustment for potential confounders was planned in the event of a statistically significant correlation.

Correction for multiple testing was not planned for either the prognostic or predictive analyses.

## 3 Results

### 3.1 Patient cohort and NGS analysis

In total, 112 consecutive patients with mTNBC were identified. After excluding 15 patients due to absence of genomic and clinical data, 97 patients with 165 tumor samples in total remained for evaluation (Fig. 1a). Out of the 165 tumor samples that were retrieved for NGS analysis, 47 samples (28 %) could not be analyzed due to insufficient quantity/quality of tumor tissue. The majority (57 %) of retrieved non-primary tumor samples (metastases and local recurrences) failed analysis whereas the failure rate of primary tumor samples was 12 % (Fig. 1b).

**Fig. 1a:**
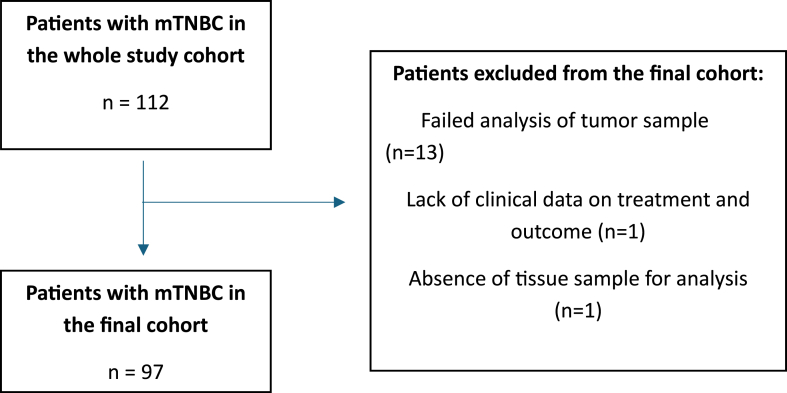
Flowchart of the study cohort.

**Fig. 1b:**
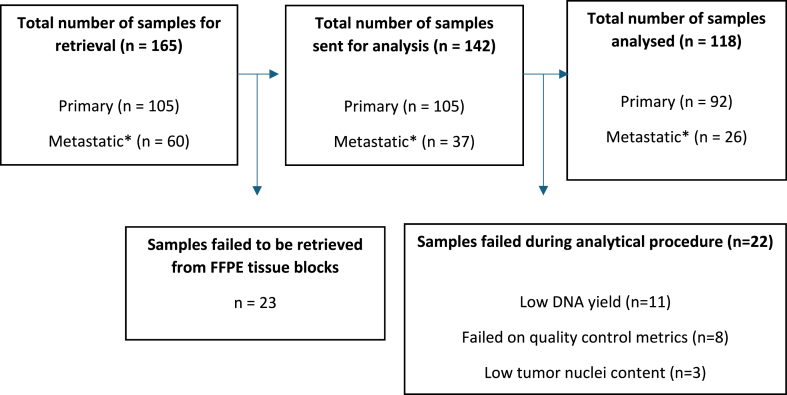
Flowchart describing the reasons for exclusion from NGS analysis of retrieved tissue samples. FFPE, Formalin-Fixed, Paraffin-Embedded. ∗ Includes samples from both metastatic tissue and local recurrences.

The median age at diagnosis of mTNBC was 61 (range: 28–80), 17 % of patients had *de novo* mTNBC and 80 % had visceral metastases. In total, 9 % of patients had an Eastern Cooperative Oncology Group Performance Status (ECOG PS) of 2 or worse. The most common chemotherapy regimens in the first-line setting were taxane-based (29 %), platinum-based (26 %), and anthracycline-based (21 %) (Table 1). Regarding modern treatment strategies, none of the patients were treated with immune checkpoint inhibitors or antibody-drug conjugates. One patient was treated with a PARP inhibitor. The median time from diagnosis of early breast cancer to metastatic relapse in patients without *de novo* mTNBC was 23.8 months.

**Table 1:** Baseline characteristics of the study cohort.

Characteristic	N (%)
**Number of patients**	97
**Age at mTNBC diagnosis, median (range), in years**	61 (28–90)
**De novo or recurrent mTNBC**	
**De novo**	16 [17]
**Recurrent**	81 (83)
**Chemotherapy for early breast cancer**^a^	
**Any chemotherapy**	52 (76)
**Neoadjuvant chemotherapy**	6 [9]
**Adjuvant chemotherapy**	46 (68)
**Taxane-based**	41 (60)
**Anthracycline-based**	45 (66)
**Platinum-based**	3 [4]
**Capecitabine-based**	5 [7]
**Metastatic sites**	
**Lymph node**	48 (49)
**Bone**	28 [29]
**Liver**	32 [33]
**Lungs**	49 (51)
**Central nervous system**	18 [19]
**Skin**	10 [10]
**Any visceral organ**	78 (80)
**ECOG PS at start of first-line chemotherapy**	
**0**	43 (44)
**1**	26 [27]
**2**	7 [7]
**3**	2 [2]
**Unknown**	19 [20]
**Chemotherapy first line**	
**Taxane-based**	28 [29]
**Anthracycline-based**	20 [21]
**Platinum-based**^b^	25 [26]
**Capecitabine-based**	14 [14]
**Other**	6 [6]
**None**	4 [4]
**Lines of systemic therapy for mTNBC, median (range)**	3 (0–10)

*Abbreviations***:** mTNBC, metastatic triple negative breast cancer; ECOG PS, Eastern Cooperative Oncology Group Performance Status.

a Among patients who received any chemotherapy for mTNBC.

b Any platinum-based regimen even if including for example a taxane.

### 3.2 Frequency of genomic alterations

**Fig. 2:**
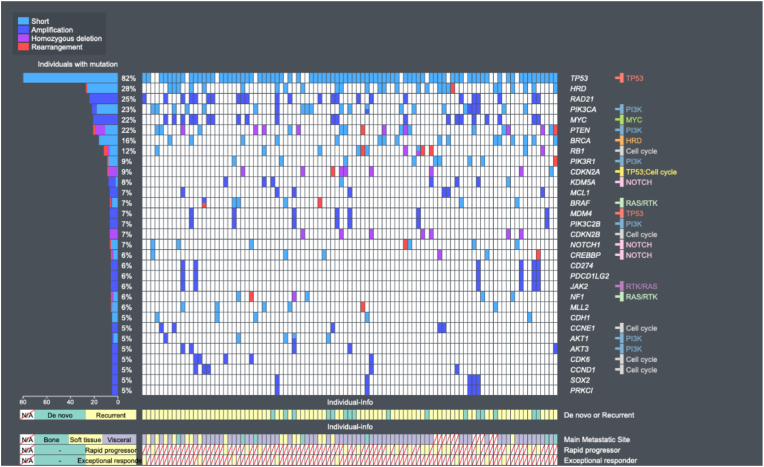
The genomic landscape of the 97-patient cohort. Gene alterations are correlated with the corresponding involved oncogenic signaling pathways (to the far right in the figure) and clinical characteristics. Gene alterations with a prevalence of less than 5 % are excluded. All gene alterations that lead to HRD were collected into one group and BRCA1 and BRCA2 mutations were combined into one group (BRCA).

The most frequently altered gene in the population was TP53 (82 %) followed by RAD21 (25 %) and PIK3CA (23 %). The prevalence of tBRCA was 16 % and the total prevalence of genomic alterations that lead to HRD was 28 %. The most frequently altered oncogenic signaling pathways were: TP53 (86 %), PI3K (60 %) and RTK/RAS (47 %) (Fig. 2). Subgrouping of HRD into specific alterations are detailed in the supplementary appendix (Table S3).

### 3.3 Tumor mutational burden and microsatellite instability

The median tumor mutational burden (TMB) was 2.52 mutations/megabase and 5 patients had a TMB of 10 or higher. None of the patients exhibited microsatellite instability.

### 3.4 Prognostic role of genomic alterations

We found no statistically significant correlation between any genomic alteration and OS (Table 2). When stratifying the cohort based on F1CDx cutoffs for TMB: TMB low (≤5 mutations per megabase) vs intermediate (>5 to < 20 mutations per megabase) or high (20 ≤ mutations per megabase), no correlation with OS was observed (HR 1.38; 95 % CI 0.84–2.29).

**Table 2:** Association between the most common genomic alterations and overall survival (OS).

Genomic alteration	Unadjusted HR	95 % CI
**TP53**	1.03	0.60–1.75
**HRD**	0.82	0.51–1.31
**BRCA**	0.59	0.37–1.07
**RTK-RAS pathway**	1.01	0.66–1.55
**PI3K pathway**	0.83	0.54–1.27
**MYC pathway**	1.11	0.67–1.83
**Cell Cycle pathway**	1.40	0.90–2.18
**NOTCH pathway**	0.80	0.49–1.31

*Abbreviations:* HRD, Homologous Recombination Deficiency; HR, Hazard Ratio; CI, Confidence Interval.

### 3.5 Predictive role of genomic alterations to chemotherapy

A lower risk of progression or death was observed for patients with tBRCA (HR 0.33; 95 % CI: 0.12–0.92) and HRD (HR 0.32; 95 % CI: 0.12–0.84) who received platinum-based chemotherapy in the first-line setting compared to patients without tBRCA and HRD. There were no other alterations predictive for rwPFS with taxane-based, anthracycline-based, or platinum-based chemotherapy in the first-line setting (Table 3).

**Table 3:** Real-world progression-Free Survival (rwPFS) on specific chemotherapeutic regimens as first-line treatment based on different genomic alterations. Patients with or without the studied genomic alterations who received the same type of chemotherapy regimen in the first line were compared.

Genomic alteration	Type of chemotherapy	Unadjusted HR	95 % CI
**TP53**	Taxane-based	1.61	0.70–3.70
	Anthacycline-based	0.78	0.22–2.81
	Platinum-based	NC	NC
**HRD**	Taxane-based	1.33	0.62–2.84
	Anthacycline-based	1.07	0.40–2.87
	Platinum-based	0.31	0.12–0.84
**BRCA**	Taxane-based	1.65	0.64–4.31
	Anthacycline-based	1.34	0.43–4.21
	Platinum-based	0.33	0.12–0.92
**RTK-RAS pathway**	Taxane-based	0.90	0.46–1.73
	Anthacycline-based	0.45	0.15–1.32
	Platinum-based	1.08	0.48–2.45
**PI3K pathway**	Taxane-based	1.20	0.62–2.35
	Anthacycline-based	1.64	0.62–4.32
	Platinum-based	0.63	0.27–1.44
**MYC pathway**	Taxane-based	0.84	0.40–1.85
	Anthacycline-based	0.94	0.26–3.40
	Platinum-based	0.52	0.19–1.43
**Cell Cycle pathway**	Taxane-based	1.09	0.55–2.12
	Anthacycline-based	2.20	0.83 – 5-84
	Platinum-based	0.94	0.40–2.22
**NOTCH pathway**	Taxane-based	1.06	0.52–2.17
	Anthacycline-based	0.46	0.10–2.02
	Platinum-based	1.71	0.67–4.35

*Abbreviations*: NC, not calculated; HRD, Homologous Recombination Deficiency; HR, Hazard Ratio; CI, Confidence Interval.

The predictive role of genomic alterations in determining the effectiveness of different chemotherapy regimens, as expressed by real-world RR in any treatment line is detailed in Table S1 in the supplementary appendix. This analysis did not reveal a statistically significant differential treatment effect in patients with HRD or tBRCA treated with platinum-based chemotherapy. Among patients who received Capecitabine in any treatment line, a TP53 mutation was associated with a lower probability of response (OR 0.14; 95 % CI: 0.02–0.98).

### 3.6 Exceptional responders and rapid progressors to chemotherapy

Patients with an exceptional response to chemotherapy were numerically younger and had a lower Charlson comorbidity index than patients who were defined as rapid progressors (Table 4). None of the exceptional responders had *de novo* metastatic disease compared to 28 % of the rapid progressors. Mutations in BRCA, MYC and the MYC pathway as well as the RAS/RTK pathway were numerically more frequent in exceptional responders whereas alterations in the Cell cycle pathway were numerically more frequent in rapid progressors. An overview of treatment regimens and responses from the 1st through 4th line of treatment among exceptional responders stratified by presence of tBRCA and HRD are provided in the supplementary appendix (Table S2).

**Table 4:** Clinical and genomic characteristics of exceptional responders and rapid progressors to chemotherapy.

Characteristic	Exceptional responder N = 8 (%)	Rapid progressor N = 18 (%)	P value
**Clinical data**			
**Visceral disease**	7 (88 %)	17 (94 %)	0.529
**Age at diagnosis of mTNBC, median (range), in years**	51.5 (35–67)	66.5 (33–82)	0.202
**Charlson comorbidity index, median (range)**	0.5 (0–2)	3 (0–8)	0.015
**ECOG PS 0**–**1 at start of first-line chemotherapy**	6 (75)	10 (72)	0.278
**Platinum-based chemotherapy for mTNBC**	3 (38)	5 [28]	0.667
**De novo mTNBC**	0 (0)	5 [28]	0.281
**Genomic alterations**			
**TP53**	7 (88)	15 (83)	1.000
**PIK3CA**	2 [25]	3 [17]	0.628
**BRCA**	2 [25]	2 [11]	0.563
**MYC**	4 (50)	3 [17]	0.149
**RAD21**	3 (38)	4 [22]	0.635
**HRD**	2 [25]	4 [22]	1.000
**Altered oncogenic pathways**			
**TP53**	7 (88)	16 (89)	1.000
**PI3K**	5 (63)	9 (50)	0.683
**MYC**	4 (50)	4 [22]	0.197
**NOTCH**	3 (38)	6 [33]	1.000
**Cell cycle**	2 [25]	9 (50)	0.395
**RAS/RTK**	5 (63)	6 [33]	0.218
**Number of pathways altered, median (range)**	3 (1–3)	3 (1–3)	0.978

*Abbreviations***:** mTNBC, metastatic triple negative breast cancer; ECOG PS, Eastern Cooperative Oncology Group Performance Status; HRD, Homologous Recombination Deficiency.

## 4 Discussion

The present study applied tumor-based comprehensive genomic profiling to a cohort of consecutive patients with mTNBC treated in a real-world setting to explore the potential prognostic or predictive value of common genomic alterations. Although a prognostic role of specific genomic alterations or tumor mutational burden could not be confirmed, some predictive value of pathogenic variants of tBRCA and genomic alterations in HRD-related genes for platinum-based chemotherapy was implied. In addition, our findings highlight the possibility of comparing the molecular profile of exceptional responders and rapid progressors for hypothesis-generating observations, since genomic alterations in BRCA, MYC, and RAS/RTK pathways were more frequent in exceptional responders and alterations in the Cell cycle pathway were more frequent in rapid progressors.

Consistent with previous studies including patients with mTNBC [7,8], TP53 was the most frequently altered gene in our study population. A potential prognostic role of TP53 alterations was not observed in our study cohort that is in contrast with some studies^11,12^ but in accordance with others^10^ thus highlighting the difficulties in validating potential prognostic biomarkers across different studies.

The lack of association between genomic alterations in specific genes or oncogenic signaling pathways and prognosis should be interpreted with caution considering the limited sample size of this study. Our approach of subdividing genes into canonical oncogenic signaling pathways can be considered as a way of circumventing the problem of small sample size, though with risk of missing prognostic information for specific genomic alterations within pathways.

Regarding genomic alterations with potential predictive role to chemotherapy, our findings suggest some associations that deserve attention. Firstly, the predictive role of tBRCA in relation to treatment with platinum-based chemotherapy seen in previous studies [19,22] was indicated by in this real-world cohort. In addition, the suggested association between HRD status and rwPFS in patients treated with platinum-based chemotherapy in the first-line setting is in line with previous studies [[22], [23], [24]], but in contrast to the TNT trial where an association between HRD and improved overall response rate in patients treated with Carboplatin compared to Docetaxel was absent [19]. Current evidence on the predictive role of HRD is complicated by the varying definitions of HRD, which makes comparisons among studies and generalizability of results challenging. In fact, the TNT trial defined HRD as either [1] presence of a high dichotomized HRD score according to the Myriad assay or [2] presence of a BRCA1/2 mutation or in the absence of a BRCA 1/2 mutation, a high HRD score. Furthermore, a cohort of patients in the ProfiLER-01 trial with a BRCA1-or RAD51C promoter methylation responded unfavorably to platinum-based chemotherapy^20^ implying that promoter methylation of HRD-related genes might not lead to similar loss-of-function as other genomic alterations in the same genes. We utilized a previously proposed pragmatic approach for HRD definition that does not require any additional genomic analyses beyond comprehensive genomic profiling. This is especially valuable given the insufficient quality/quantity of tumor tissue in real-world settings as highlighted in our study. One limitation with our HRD cohort was that only 12 out of 28 patients had HRD without tBRCA and among this subgroup only 2 patients received platinum-based chemotherapy at any time. This means that the predictive role of HRD for platinum-based chemotherapy almost exclusively was attributable to the tBRCA subgroup.

Our finding that TP53-mutated tumors responded unfavorably to Capecitabine is consistent with the findings reported by Tian et al. [32] where TP53 mutations were overrepresented in a group of patients with short PFS on Capecitabine maintenance therapy. However, due to the small sample size of our study and the absence of multivariate analyses in both studies these findings warrant further confirmation in larger prospective trials.

The explorative comparison between patients that exhibited an exceptional response and rapid progression to chemotherapy can potentially provide a basis for generating hypotheses for future studies of molecular biomarkers in mTNBC. We observed some patterns of overrepresentation of certain genomic alterations, such as MYC and RAS/RTK pathways in the exceptional response group and Cell cycle pathway in rapid progressors. Further investigation of this approach in larger cohorts has the potential to uncover new predictive biomarkers by exposing genomic vulnerabilities and mechanisms of resistance to different treatment strategies when the treatment outcome is an outlier.

This study has several limitations that might impact the internal validity and generalizability of study results. Firstly, the study design limits the possibility to establish any causal inference between genomic alterations and treatment response to specific chemotherapy agents and the results should be considered as hypothesis-generating. Considering the nature of the study, we chose not to adjust our analyses for multiple testing thus increasing the risk of type I errors. However, this approach enables broader explorative analyses that are suitable for the hypothesis-generating nature of the study. Furthermore, the relatively modest sample size limits the statistical power to detect potential associations of less frequent genomic alterations with prognosis and treatment response.

Additionally, this study population was treated before the advent of antibody-drug conjugates (ADCs) and immune checkpoint inhibitors in mTNBC. Given the significant development of ADCs in mTNBC and the sparsity of biomarkers in this treatment strategy, similar studies investigating genomic predictive biomarkers for ADCs would be valuable. Our study also lacked data on programmed death-ligand 1 (PD-L1) expression which could be studied as a predictive biomarker for chemotherapy as well.

Furthermore, our study lacked data regarding germline BRCA status, making it unknown what proportions of the tBRCA mutations observed were germline derived versus somatically acquired. One concern with using tBRCA as a predictive biomarker for DNA-damaging agents has been that not all mutations may exhibit loss of heterozygosity (LOH), thus not rendering the cell vulnerable to DNA damage. However, several studies [[33], [34], [35]] have shown that the vast majority of tBRCA exhibit LOH. Thus, there is in addition to the clinical data demonstrated in our study, also a biological rationale for using tBRCA as a predictive biomarker.

Finally, all analyses were mainly based on genomic data from the primary tumor tissue. The choice to prioritize the genomic data obtained from primary tumor tissue samples for the analyses even when metastatic tissue samples were available, aimed to reduce the heterogeneity of genomic data. Although discordance of genomic alterations may be exhibited between primary tumor and metastatic tissue samples, we consider our approach feasible based on data of paired samples from patients with TNBC, indicating that genomic alterations are largely retained during disease progression [36,37].

## 5 Conclusion

Our study results on genomic characterization of TNBC in a real-world setting give some hypothesis-generating insights on the potential prognostic and predictive roles of genomic alterations. We could not confirm any prognostic role of TP53 or other common genomic alterations in mTNBC. However, our study reinforces the growing body of evidence supporting tBRCA and HRD as potential predictive biomarkers for platinum-based chemotherapy in patients with mTNBC. A potential negative impact of TP53 mutations on the response to Capecitabine is implied, but further exploration is needed. Finally, our research highlights the possibility of exploring the genomic profile of exceptional responders and rapid progressors as a potential approach for identifying new molecular biomarkers.

## CRediT authorship contribution statement

**Erik Olsson:** Writing – review & editing, Writing – original draft, Visualization, Methodology, Investigation, Formal analysis, Data curation, Conceptualization. **Henrik Lindman:** Writing – review & editing, Supervision, Project administration, Methodology, Investigation, Funding acquisition, Formal analysis, Data curation, Conceptualization. **Evangelos Digkas:** Writing – review & editing, Methodology, Data curation, Conceptualization. **Viktoria Thurfjell:** Writing – review & editing, Investigation, Data curation, Conceptualization. **Haidar Mir Ali:** Writing – review & editing, Methodology, Data curation, Conceptualization. **Ute Krüger:** Writing – review & editing, Methodology, Investigation, Data curation. **Anna-Karin Wennstig:** Writing – review & editing, Methodology, Data curation. **Marie Sundqvist:** Writing – review & editing, Methodology, Data curation. **Antonios Valachis:** Writing – review & editing, Writing – original draft, Visualization, Supervision, Project administration, Methodology, Funding acquisition, Formal analysis, Data curation, Conceptualization.

## Data statement

The datasets generated during and/or analyzed during the current study are not publicly available due to ethical reasons but are available from the corresponding author upon reasonable request.

## Ethical approval

The study received ethical approval from the Swedish Ethical Review Authority (reference number: 2017/584-31 and 2019–06011) and was conducted in accordance with the declaration of Helsinki.

## Declaration of competing interest

The authors declare that they have no known competing financial interests or personal relationships that could have appeared to influence the work reported in this paper.
