# Supplementary material for: Genomic characterization and molecular predictive biomarkers for chemotherapy in patients with metastatic triple-negative breast cancer treated in a real-world setting
Source: Breast. 2025 Jan 2;79:103874. doi: 10.1016/j.breast.2025.103874 (PMC11761257; doi:10.1016/j.breast.2025.103874)
Supplement: Multimedia component 1 [file mmc1.docx]

**Supplementary appendix**

**Table S1:** Real-world response rate on specific chemotherapeutic regimens given in any line based on different genomic alterations. Patients with or without the studied genomic alteration who received the same type of chemotherapy were compared.

| **Genomic alteration** | **Type of chemotherapy** | **Unadjusted OR** | **95% CI** |
| --- | --- | --- | --- |
| **TP53** | Taxane-based  Anthacycline-based  Platinum-based  Capecitabine-based | 0.67  NC  NC  0.14 | 0.14 – 3.19  NC  NC  0.02 – 0.98 |
| **HRD** | Taxane-based  Anthacycline-based  Platinum-based  Capecitabine-based | 0.62  2.00  1.20  0.17 | 0.13 – 2.95  0.43 – 9.27  0.27 – 5.40  0.02 – 1.61 |
| **BRCA** | Taxane-based  Anthacycline-based  Platinum-based  Capecitabine-based | 0.30  2.25  1.25  0.40 | 0.03 – 2.98  0.33 – 15.54  0.26 – 6.07  0.04 – 3.96 |
| **RTK-RAS pathway** | Taxane-based  Anthacycline-based  Platinum-based  Capecitabine-based | 2.91  1.50  0.49  1.38 | 0.77 – 11.07  0.39 – 5.18  0.11 – 2.16  0.31 – 6.20 |
| **PI3K pathway** | Taxane-based  Anthacycline-based  Platinum-based  Capecitabine-based | 1.39  2.79  4.19  0.35 | 0.37 – 5.17  0.70 – 11.10  0.82 – 21.34  0.08 – 1.66 |
| **MYC pathway** | Taxane-based  Anthacycline-based  Platinum-based  Capecitabine-based | 0.38  0.75  0.75  0.71 | 0.07 – 2.20  0.15 – 3.79  0.14 – 4.17  0.12 – 4.32 |
| **Cell cycle pathway** | Taxane-based  Anthacycline-based  Platinum-based  Capecitabine-based | 0.40  0.33  1.53  0.57 | 0.10 – 1.64  0.08 – 1.35  0.31 – 7.44  0.10 – 3.41 |
| **NOTCH pathway** | Taxane-based  Anthacycline-based  Platinum-based  Capecitabine-based | 1.21  1.50  0.55  0.98 | 0.30 – 4.98  0.34 – 6.56  0.10 – 2.89  0.19 – 4.94 |

*Abbreviations*: NC, not calculated; HRD, Homologous Recombination Deficiency; HR, Hazard Ratio; CI, Confidence Interval.

**Table S2:** Treatment and response to 1^st^ through 4^th^ line treatment among exceptional responders. Presence of tBRCA and HRD are noted and cells are color-coded based on best response to treatment: dark green = complete response, light green = partial response, yellow = stable disease, red = progressive disease.

| **Pat ID** | **tBRCA** | **HRD** | **1st line** | **2nd line** | **3rd line** | **4th line** |
| --- | --- | --- | --- | --- | --- | --- |
| 1 | No | No | FEC | P | V | LDox |
| 2 | No | No | C+D | V |  |  |
| 3 | No | No | Cap | V | Cap+V | P |
| 4 | Yes | Yes | C |  |  |  |
| 5 | Yes | Yes | C+P | Cap | O | E |
| 6 | No | No | D | FEC | FEC | LDox |
| 7 | No | No | FEC | Other |  |  |
| 8 | No | No | P |  |  |  |

*Abbreviations*: tBRCA, tumor BRCA1/2 mutation; HRD, homologous recombination deficiency; FEC, Fluorouracil, Epirubicin and Cyclophosphamide; C, Carboplatin; D, Docetaxel; Cap, Capecitabine; O, Olaparib; P, Paclitaxel; V, Vinorelbin; E, Eribulin; LDox, Liposomal Doxorubicin.

**Table S3:** Frequency of HRD-related gene alterations. Note that one tissue sample may have more than one HRD-related gene alteration.

| **Genomic alteration** | **Frequency** |
| --- | --- |
| **BRCA1/2** | 16 |
| **Non-BRCA1/2** | 11 |
| **BRCA1** | 13 |
| **BRCA2** | 4 |
| **ARID1A** | 3 |
| **CHEK2** | 3 |
| **BARD1** | 1 |
| **FANCC** | 1 |
| **NBN** | 1 |
| **PALB2** | 1 |
| **MRE11A** | 1 |
| **ATM** | 1 |
| **BAP1** | 1 |
